# Multiple-model GWAS identifies optimal allelic combinations of quantitative trait loci for malic acid in tomato

**DOI:** 10.1093/hr/uhad021

**Published:** 2023-02-14

**Authors:** Wenxian Gai, Fan Yang, Liangdan Yuan, Saeed ul Haq, Yaru Wang, Ying Wang, Lele Shang, Fangman Li, Pingfei Ge, Haiqiang Dong, Jinbao Tao, Fei Wang, Xingyu Zhang, Yuyang Zhang

**Affiliations:** National Key Laboratory for Germplasm Innovation and Utilization of Horticultural Crops, Huazhong Agricultural University, Wuhan 430070, China; College of Horticulture, Northwest A&F University, Yangling 712100, China; National Key Laboratory for Germplasm Innovation and Utilization of Horticultural Crops, Huazhong Agricultural University, Wuhan 430070, China; College of Horticulture, Northwest A&F University, Yangling 712100, China; Department of Horticulture, The University of Agriculture Peshawar, Peshawar 25130, Pakistan; National Key Laboratory for Germplasm Innovation and Utilization of Horticultural Crops, Huazhong Agricultural University, Wuhan 430070, China; National Key Laboratory for Germplasm Innovation and Utilization of Horticultural Crops, Huazhong Agricultural University, Wuhan 430070, China; National Key Laboratory for Germplasm Innovation and Utilization of Horticultural Crops, Huazhong Agricultural University, Wuhan 430070, China; National Key Laboratory for Germplasm Innovation and Utilization of Horticultural Crops, Huazhong Agricultural University, Wuhan 430070, China; National Key Laboratory for Germplasm Innovation and Utilization of Horticultural Crops, Huazhong Agricultural University, Wuhan 430070, China; National Key Laboratory for Germplasm Innovation and Utilization of Horticultural Crops, Huazhong Agricultural University, Wuhan 430070, China; National Key Laboratory for Germplasm Innovation and Utilization of Horticultural Crops, Huazhong Agricultural University, Wuhan 430070, China; National Key Laboratory for Germplasm Innovation and Utilization of Horticultural Crops, Huazhong Agricultural University, Wuhan 430070, China; National Key Laboratory for Germplasm Innovation and Utilization of Horticultural Crops, Huazhong Agricultural University, Wuhan 430070, China; National Key Laboratory for Germplasm Innovation and Utilization of Horticultural Crops, Huazhong Agricultural University, Wuhan 430070, China; Hubei Hongshan Laboratory, Wuhan 430070, China

## Abstract

Malic acid (MA) is an important flavor acid in fruits and acts as a mediator in a series of metabolic pathways. It is important to understand the factors affecting MA metabolism for fruit flavor improvement and to understand MA-mediated biological processes. However, the metabolic accumulation of MA is controlled by complex heredity and environmental factors, making it difficult to predict and regulate the metabolism of MA. In this study, we carried out a genome-wide association study (GWAS) on MA using eight milestone models with two-environment repeats. A series of associated SNP variations were identified from the GWAS, and 15 high-confidence annotated genes were further predicted based on linkage disequilibrium and lead SNPs. The transcriptome data of candidate genes were explored within different tomato organs as well as various fruit tissues, and suggested specific expression patterns in fruit pericarp. Based on the genetic parameters of population differentiation and SNP distribution, tomato MA content has been more influenced by domestication sweeps and less affected by improvement sweeps in the long-term history of tomato breeding. In addition, genotype × environment interaction might contribute to the difference in domestication phenotypic data under different environments. This study provides new genetic insights into how tomato changed its MA content during breeding and makes available function-based markers for breeding by marker-assisted selection.

## Introduction

In ripe fruits, malic, citric, and tartaric acids are the main organic acids, and act as important indicators of the flavor and nutritional qualities of fruits. Fruit acidity is due to the presence of organic acids, and different acidic substances are not equally acidic and differ significantly in acidity [1]. Differences in total acidity or organic acid balance have also been observed in many fruits’ varieties. Malic acid (MA) is dominant in apple, pear, and loquat fruits, whereas citric acid contributes to the acidity of citrus fruits [2, 3]. In tomato, the acidity is made up of malic and citric acids, which account for >90% of the total organic acids of the harvested fruits [4]. MA is the mediator of several metabolic pathways in the mesocarp cells of fleshy fruits, such as the tricarboxylic acid cycle, the chloroplast Calvin cycle, and the glyoxalic acid cycle [2, 5]. MA is a key metabolite in maintaining osmotic pressure and charge balance, and also plays an important role in the regulation of stomatal conductance in plants [6]. Additionally, plant hormones and self-functional genes can enhance stress resistance by promoting the accumulation of MA [7, 8]. Therefore, it is of great importance to understand the genetic factors regulating (i) MA metabolism for fruit flavor improvement and (ii) MA-mediated life activities.

The MA content in fruit is determined by its synthesis anddegradation and by malate-related transporters, which are controlled by a complex molecular regulation system [9–12]. In addition to the synthesis and degradation pathways of MA representedin glycolysis and the tricarboxylic acid cycle, malate transportersplay essential roles in the final MA concentration [7, 9, 13, 14]. The transcription factor MMYB1 directly regulates the transcription ofmalate transporters *MdVHA-B*s, *MdVHA-E2*, and *MdtDT12* to control MA content in apple [14]. Many previous studies have reported that aluminum-activated malate transporter (ALMT) is involvedin malate transport, and *ALMT* mutant plants had an altered MA concentration [15, 16]. Genetic variations of *ALMT* genes have been dissected to some extent. The premature stop codon-led truncation of the apple *ALMT9* gene is genetically responsible forlow acidity in apples [13]. In tomato, the deletion of 3 bp (GTC) in the Sl-*AlMT9* promoter alters its expression, resulting in a high MA content in fruit [10]. Further studies found that the expression of *ALMT* genes is directly regulated by the transcription factorsWRKY [10] and MYB [17]. In addition, BT2 mediated the stabilityof the MYB protein through ubiquitination, which reduced theexpression of malate-related genes, thus negatively altering MA accumulation [9].

The metabolic accumulation of MA in fruit is controlled by both heredity and environment [2]. In tomato (*Solanum lycopersicum*), the MA narrow-sense heritability (*H*^2^) estimated using the tomato graph pangenome is 0.43 [18]. Some other independent estimates also revealed that the broad-sense *H*^2^ of MA ranged from 0.19 to 0.66 [3, 10, 19]. Genome-wide association studies (GWAS) provide considerable information for understanding the natural variation and genetic evolution of MA metabolism in tomato at genomic level. Researchers have recently revealed that the total heritability of MA is attributable to single-nucleotide polymorphism (SNP) variations, while insertions and deletions (indels) or structure variations (SVs) contribute less heritability using GWAS [18]. Factors such as association models, population structure, phenotype, and environmental influences all affect the results of GWAS analyses. Some single models – mixed linear model (MLM) [20, 21], multiple-locus mixed model (MLMM) [19, 22], compression MLM (CMLM) [10], and FaST-LMM models [3] – have been used to identified these significant SNP variations associated with tomato MA concentration. However, the number of identified significant association loci varied significantly between studies, ranging from a single peak SNP to dozens of peak SNPs [3, 21]. All these association results identified the most significant SNP marker associations in close physical proximity on chromosome 6, which was a major quantitative trait locus (QTL), responsible for MA accumulation in tomato fruit. Ye *et al*. (2017) found that this QTL corresponded to the *ALMT* gene (a single gene, Solyc06g072910/Solyc06g072920) and proved that the *ALMT9* gene was involved in determining the MA content in fruits [10].

In this study, we report the assessment of superior alleles controlling MA accumulation in ripe tomato fruits. The objective of this study was to identify these loci and decipher the polygenic architecture of the regulation of MA content. We carried out a GWAS using six milestone models with two-environment repeats. A series of associated SNP variations were identified from GWAS, and high-confidence annotated genes were obtained based on the lead SNPs and the MA distribution as a function of genotypes at the lead SNPs. The genetic parameters of population differentiation were employed to identify potential selective sweep signals on MA during domestication and improvement steps. This study will provide new genetic insights into how tomato MA content evolved during breeding and the optimal QTL combination for MA improvement.

**Figure 1:**
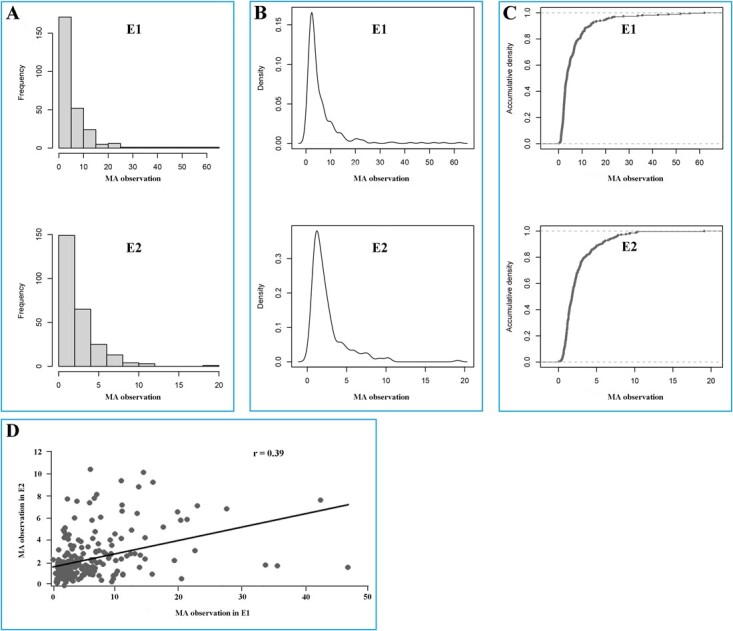
Phenotype view of MA content under two environments. Frequency distribution (A), density (B), and accumulative density of MA content (C) under E1 and E2 environments. (D) Correlation between MA levels from two different environments; *r* represents the Pearson correlation coefficient. The phenotypes of 266 (E1) and 260 (E2) tomato accessions were acquired from two independent environments and were used to perform the GWAS. A detailed description of the tomato accessions and MA content is provided in Supplementary Data Table S1.

## Results

### GWAS identifies multiple loci underlying MA content

There were broad phenotypic variations in MA content evaluated in both environments (Fig. 1, Supplementary Data Table S1), and therefore there might be a large amount of genetic variation involved in controlling fruit MA accumulation. The frequency distribution of the phenotypic values showed that a large number of accessions accumulated low MA (Fig. 1A) and conformed to approximate normal distribution characteristics (Fig. 1B). Considering the population structure and size in each phenotypic class, data were used to dissect complex characters by association analyses. The broad-sense *H*^2^ estimate for MA content was .58 based on two-repeat phenotypic data. The Pearson correlation coefficient (*r* = .39) for MA levels indicated a positive correlation of MA content in the two environments. For the combinations of phenotype and genotype, we constructed a high-density genotyping subset of 3 535 044 SNPs for subsequent analyses by filtering all SNPs with a missing rate <20% and minor allele frequencies <.05 (Supplementary Data Fig. S1C). Among these tomato accessions, the individual heterozygosity did not exceed .8, and the heterozygosity of most germplasms ranged from 0 to .1 (Supplementary Data Fig. S1A), leading to the identification of heterozygous markers in the SNP set.

Six different association mapping models that ranged from simple to complex were used to perform the GWAS. One hundred and fifty significant SNPs (*P* < 2.83 × 10^−7^) were identified from six-model results and are listed in Supplementary Data Tables S2 and S3. Considering the same thresholds, the six models recognized different numbers of suggestive SNPs associated with MA content in environment E1 (Supplementary Data Tables S2–S4). For example, if we qualified the significant threshold as −log10(*P*) > 7.85 to gain significant association SNPs, we identified 10 SNPs from BLINK (Linkage-disequilibrium Iteratively Nested Keyway), 11 from CMLM, 4 from FarmCPU, 11 from GLM, 11 from MLM, and 2 from MLMM. The associated loci and the number of them showed differences between E1 and E2, and it was inferred that the statistical power between different methods and the environmental factors contributed to these differences. More than one significant/suggestive linked SNP was identified for all models; for single-locus models in particular there were many associated SNPs in E2. A smaller number of associated SNPs from multiple-locus models were acquired than single-locus models. In addition to BLINK identifying some obvious false-positive sites in environment E1, the numbers of suggestive or significant SNPs were similar from GWAS results with different single-locus models or multiple-locus models, respectively. Additionally, single-locus models generated some large peaks consisting of many SNPs because one SNP in these peaks had the highest correlation with MA content, but the other SNPs in a given peak were at high linkage disequilibrium (LD) with the peak SNP. The number of significant loci identified by single-locus models was obviously higher than that identified by multiple-locus models (Supplementary Data Table S4). Interestingly, the multi-site model also acquired some specific loci, such as TFM7.1, which could only be detected by the FarmCPU model (Table 1). These results suggested that GWAS results might vary depending on the type of association mapping model, and the characteristics of the model itself would be very important in determining the selection of candidate genes.

**Figure 2:**
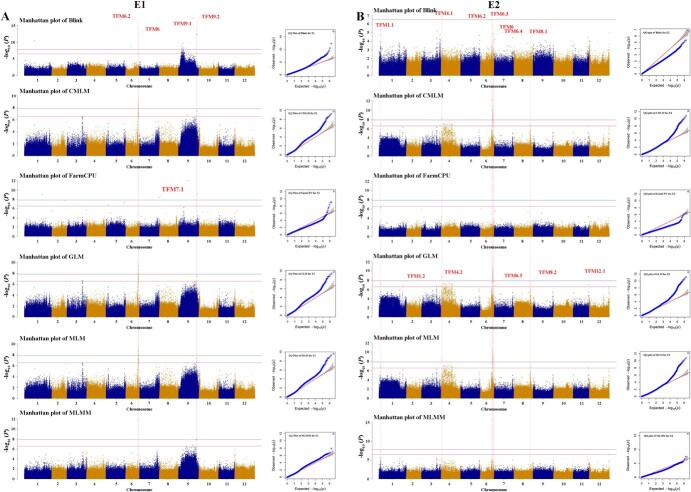
Manhattan and QQ plots for six GWAS models for E1 (A) and E2 (B). The −log10(*P*) values from the GWAS results are plotted on the *y* axis. The blue lines indicate a genome-wide significant threshold of 7.85 and the orange lines indicate a suggestive threshold of 6.55. The loci associated with candidate genes (Table 1) are marked in the Manhattan plots by red dotted lines. QQ plots are at the right of the corresponding Manhattan plots.

To understand the GWAS results, we constructed Manhattan and QQ plots (Fig. 2). QQ plots for single-locus models had a line with a strongly deviated tail, which might indicate that these models increased the false positives. For instance, significant *P*-values of SNPs ch06_41074832 (TFM6.2) and ch06_41338689 (TFM6) were detected in the GWAS results with all models except BLINK (Supplementary Data Tables S2 and S3). Many SNPs resulting from certain models (e.g. BLINK in E1 and E2, and FarmCPU in E1) were close to the straight line of 1:1, signifying that these associated SNPs might have been recognized as false negatives. For example, the SNP ch09_60583005 (TFM9.2) was detected from the FarmCPU, BLINK, CMLM, MLM, and GLM models in E1; however, no signal was present in this site from the MLMM model (Fig. 2A). Based on the GWAS results with multiple models and two environments, three types of associated lead SNPs were defined and are shown in Supplementary Data Table S2 and Table 1 (MT, multiple models and two environments; MS, multiple models and single environment; SS, single model and single environment). Among them, MT-type SNPs exhibited high association from multiple models in two environments. In MLM-model GWAS results, the SNPs ch06_41074832 and ch06_41338689 explained 16.66 and 15.41% of the phenotypic variation in E1, and explained 22.45 and 12.37% of the phenotypic variation in E2, respectively. This evidence implied that TFM6.2 and TFM6 might be the major loci associated with MA content. Multiple-model and two-environment GWAS based on high-density SNP markers could be accurate and effective for the prediction of MA regulatory genes.

**Table 1:** Summary of identified significant associations. For each locus, the SNP, chromosome (CHR), position (bp), reference allele (Ref), alternative allele (Alt), environment, model, −log10(*P*), candidate gene, and known loci are provided

Type^a^	Locus	Lead SNP	CHR	Position	Ref	Alt	Environment	Model	−log10(*P*)	Candidate gene	Identical to or close to the known SNP
MT	TFM6.2	ch06_41074832	6	41074832	T	A	E1	GLM	9.75	Solyc06g072440	[21]
								MLM	9.64		
								MLMM	11.91		
								CMLM	9.76		
							E2	CMLM	7.29		
								GLM	7.72		
								MLM	7.29		
	TFM6	ch06_41338689	6	41338689	C	T	E1	GLM	9.18	Solyc06g072910/Solyc06g072920	[3, 10, 19–22]
								MLM	9.02		
								CMLM	8.98		
							E2	CMLM	12.15		
								FarmCPU	12.76		
								GLM	12.55		
								MLMM	15.62		
								MLM	12.15		
MS	TFM1.1	ch01_5153996	1	5153996	C	G	E2	CMLM	6.90	Solyc01g010350	NA
								GLM	7.20		
								MLMM	7.40		
								MLM	6.90		
	TFM4.1	ch04_2164254	4	2164254	A	T	E2	CMLM	8.14	Solyc04g008540	[20, 21]
								GLM	8.46		
								MLM	8.14		
	TFM6.3	ch06_41008267	6	41008267	G	A	E2	CMLM	8.07	Solyc06g072340	[21]
								GLM	8.51		
								MLM	8.07		
	TFM6.4	ch06_45067556	6	45067556	A	T	E2	CMLM	7.35	Solyc06g083330	NA
								GLM	7.60		
								MLM	7.35		
	TFM8.1	ch08_53574022	8	53574022	T	A	E2	CMLM	6.64	Solyc08g067300	[20]
								GLM	6.89		
								MLM	6.64		
	TFM9.1	ch09_59796046	9	59796046	T	C	E1	MLM	6.60	Solyc09g065900	NA
								MLMM	7.88		
								CMLM	6.70		
								GLM	6.66		
	TFM9.2	ch09_60583005	9	60583005	T	C	E1	BLINK	12.29	Solyc09g072520	NA
								FarmCPU	9.05		
								MLM	7.34		
								CMLM	7.45		
								GLM	7.49		
SS	TFM1.2	ch01_78818632	1	78818632	T	C	E2	GLM	6.56	Solyc01g095910	NA
	TFM4.2	ch04_2970001	4	2970001	T	A	E2	GLM	6.72	Solyc04g009560	NA
	TFM6.5	ch06_43832543	6	43832543	G	A	E2	GLM	6.59	Solyc06g076360	[21]
	TFM7.1	ch07_63453334	7	63453334	G	C	E1	FarmCPU	8.38	Solyc07g063870	NA
	TFM8.2	ch08_53462894	8	53462894	C	T	E2	GLM	6.89	Solyc08g067210	[21]
	TFM12.1	ch12_64414473	12	64414473	A	T	E2	GLM	6.56	Solyc12g098630	NA

a Multiple models and two environments (MT), multiple models and single environment (MS), single model and single environment (SS). NA represents none.

**Figure 3:**
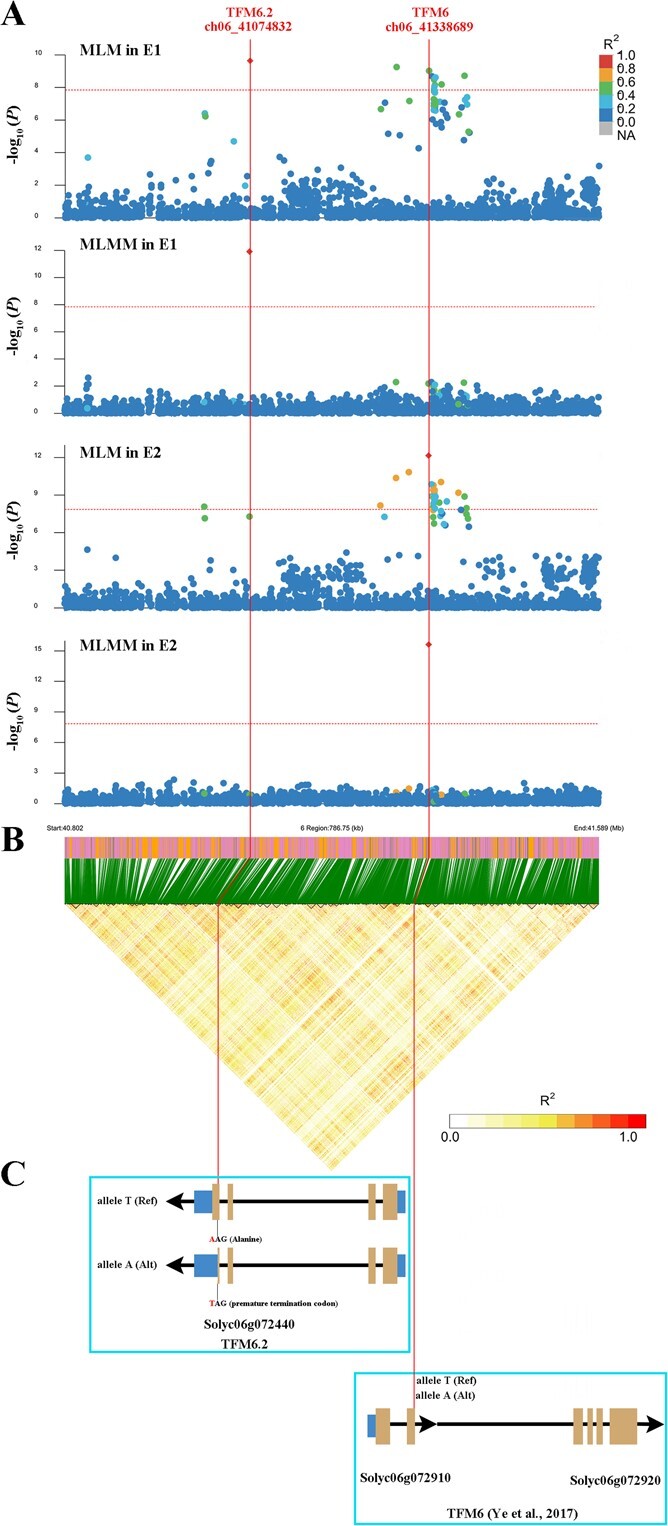
Identification and analyses of major loci associated with MA content. The dotted red lines through the figure denote the SNPs ch06_41074832 and ch06_41338689. (A) Detailed plots selected from representative GWAS results in region 40.8–41.59 Mb on chromosome 6 (*x*-axis). Lead SNPs in each plot are indicated in red. The pairwise *R*^2^ values among all SNPs are given in the color scale. The solid red lines indicate the significance threshold of the *P*-value (1.41 × 10^−8^). (B) Heat map depicting the LD block in the 0.79 M genomic region corresponding to (A). The LD blocks within this region are indicated with a black border. The *R*^2^ values are indicated in the color scale. Magenta and green vertical lines represent SNPs located in LD blocks and orange vertical lines represent no SNP. The green slashes indicate the position of the SNPs in the LD blocks. (C) Candidate gene models. For each gene model, blue boxes represent untranslated regions, yellow boxes represent coding sequences, thin black lines between boxes represent introns, and thick black arrows indicate gene orientation. The T to A change in the forward strand DNA converts an AAG codon (Gln) to a TAG in the reverse strand (coding DNA strand), causing premature termination of translation. A previous study has proved that Solyc06g072910 and Solyc06g072920 are a single gene [10].

### Candidate genes for MA accumulation in tomato

**Figure 4:**
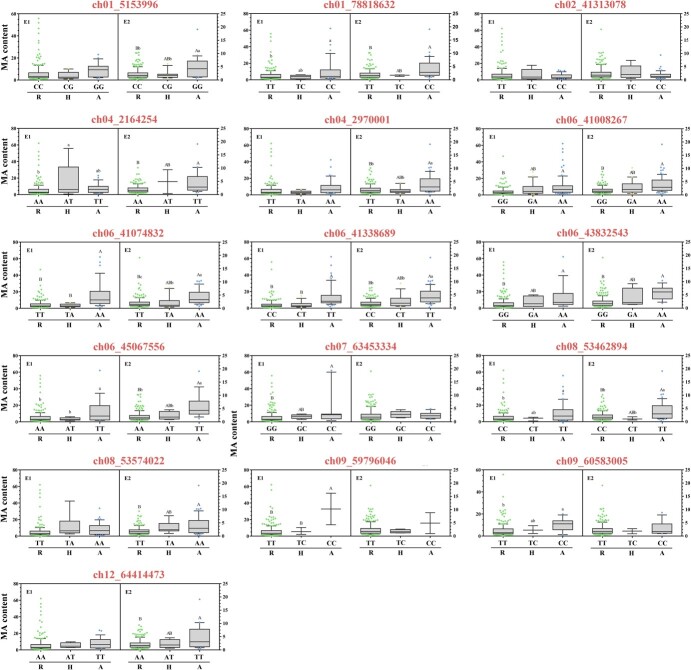
Comparative analyses of MA content in accessions with different lead SNP genotypes. Based on the variation in position of association SNPs, we analyzed the distributions as a function of 16 SNP genotypes. Different uppercase or lowercase letters represent significant differences at *P* ≤ .0001or .01 by *t*-test, respectively. R represents the homozygous reference allele; A represents a homozygous alternative allele; H represents a heterozygous allele.

One of the key goals of GWAS is the accurate identification and utilization of causal genes underlying agronomically important traits. Two environments were suitable to identify major/fixed SNPs (MT type) and environment-effect SNPs (MS and SS types) (Table 1). Most significant SNPs were in an independent LD block, except that TFM6 has high *R*^2^ (0.6–1 MLM in E2) with other significant SNPs (Fig. 3A and B). QTL TFM6 was detected in both environments, but showed different lead SNPs with high LD value (*R*^2^ = 0.6–0.8) (Fig. 3A, Supplementary Data Fig. S2). This also showed the necessity of multi-model and multi-environment GWAS analysis. Considering the results of two replications and six models, SNP ch06_41338689 was recognized as the lead SNP of TFM6. Moreover, QTLs (TFM6 and TFM6.2) were repeatedly detected across the two years, indicating the roles of their major QTLs (Figs 2 and 3, Table 1). Based on the variation in position of association SNPs (Table 1, Supplementary Data Table S2) and LD block (Fig. 3), 16 lead SNPs located at the genes or gene promoters (ch08_53574022) were used to predict directly linked genes. In an association study, lead SNPs might have a stronger linkage with MA content, and thus we analyzed the distribution of MA as a function of genotypes at the 16 associated SNPs (Fig. 4). Due to incompletely homozygous individuals (Supplementary Data Fig. S1A), the genotypes of all 15 loci were divided into three types (R, H, and A). Only SNP chr02_41313078 displayed no MA content difference between the three genotypes, signifying that it did not effectively distinguish between high- and low-MA accessions, and was not used to predict candidate genes. Finally, 15 candidate genes related to MA content were obtained and are summarized in Table 1.

Each genotype corresponded to a different level of MA content. Genotype analyses of 15 candidate gene-related SNPs (except chr02_41313078) showed that all the genotypes R (the genotype of the reference genome) were alleles related to low MA levels (Fig. 4), suggesting that the reference accession (*S. lycopersicum* cv. ‘Heinz’) might be lacking MA individuals. The MA content of genotype H was between those of genotypes A and R, and there was no additive effect of lead loci. The partial H genotypes showed no differences from genotypes R and A, such as chr01_78818632, chr06_41008287, and chr08_53574022. Moreover, chr06_45067556 and chr06_41338689 genotypes R were completely dominant in both E1 and E2, while chr02_2164254 genotype A also showed a dominant effect in E1. It was worth noting that chr06_41074832 R showed complete dominance in E1 and incomplete dominance in E2, and the environment factor was an uncertainty that might affect the outcome. In addition, the distribution of MT-type SNP alleles (Table 1) exhibited significant differences among the high- and low-MA cultivars. Although significant signals of MS-type SNP alleles (e.g. TFM4.1, TFM6.3, and TFM6.4) or SS-type SNP alleles (e.g. TFM1.2, TFM7.1, and TFM8.2) were detected from different models in E2, the distributions of their lead SNP alleles were significantly different in tomato fruits differing in MA content. These results suggested that detailed SNP genotyping played an auxiliary role in identifying candidate genes, and could evaluate the genetic effect of SNPs on MA content.

**Figure 5:**
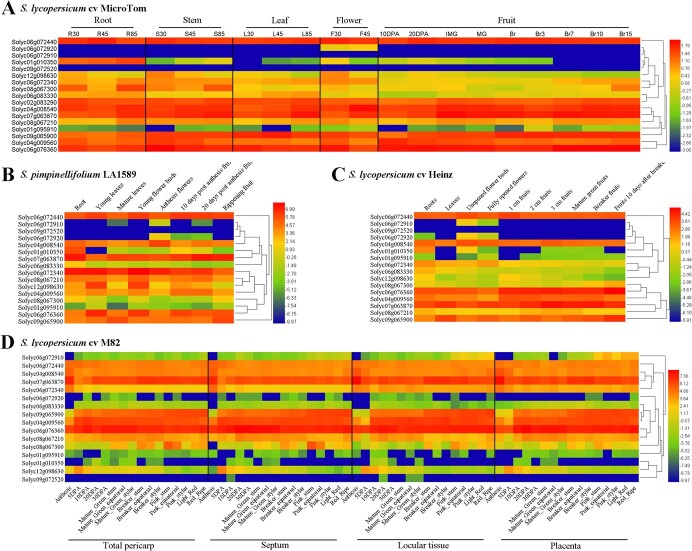
Expression patterns of candidate genes. The candidate gene expressions of different tomato organs from *S. lycopersicum* cv. ‘Micro-Tom’ (TS-7) (A), *S. pimpinellifolium* LA1589 (TS-19) (B), and *S. lycopersicum* cv. ‘Heinz’ (reference genome) (C), and different fruit tissues from *S. lycopersicum* cv. ‘M82’ (TS-3/228) (D) are displayed as heat maps. The expression levels are normalized using log10. Genes are clustered according to their expression patterns.

In an attempt to explore whether these candidate genes have fruit-specific expression patterns, we collected the transcript data from several public databases (Fig. 5). We analyzed expressions in different tomato organs (Fig. 5A–C). Although Solyc06g072910 and Solyc06g072920 have been proved to be a single gene [10], their tissue-specific expressions also showed differences. For example, Solyc06g072920 was specifically expressed in ‘Micro-Tom’ flowers (Fig. 5A) and ‘Heinz’ roots (Fig. 5C), and showed higher expression levels than Solyc06g072910 in anthesis flowers and 10- and 20-day postanthesis fruits of LA1589. Therefore, the possibility of alternative splicing could not be rejected for the Solyc06g072910/Solyc06g072920 gene. Some genes were specifically expressed in different organs; for instance, Solyc01g010350 showed a higher transcriptional level in roots (Fig. 5A–C) and the expressions of Solyc06g083330 and Solyc06g072340 in fruit were lower than those in other tissues (Fig. 5A and C). The expressions in different fruit tissues were also analyzed, and most genes showed little differences among total pericarp, septum, locular tissue, and placenta (Fig. 5D). It was interesting that Solyc06g072910 and Solyc06g072920 transcription patterns showed a significant difference, supporting the possibility of alternative splicing. Compared with the high accumulation of Solyc06g072440 mRNA, Solyc06g072910/Solyc06g072920 showed a lower expression level (Fig. 5). The cluster results in heat maps showed that the Solyc06g072440 branch was closed to the branches of Solyc06g072910 and Solyc06g072920, revealing similar expression patterns. Because the genotypes of SNPs ch06_41074832 (Solyc06g072440) and ch06_41338689 (Solyc06g072910/Solyc06g072920) in three germplasm materials were all RR, both alleles of low MA content, we inferred that Solyc06g072910/Solyc06g072920 positively regulated MA accumulation and Solyc06g072440 played a negative role in accumulating MA.

### Selective sweeps of MA-associated SNPs during domestication and improvement

**Figure 6:**
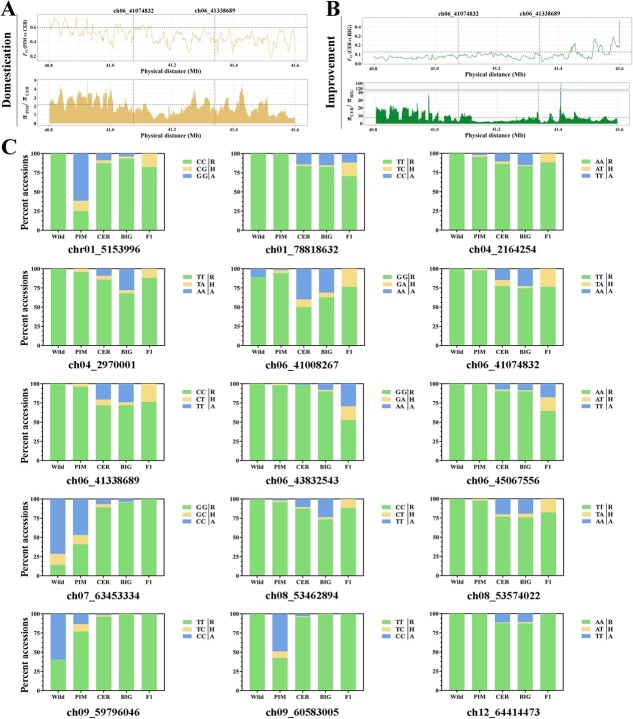
Evolution of lead SNPs during domestication and improvement. (A) Domestication and (B) improvement sweeps in region 40.8–41.59 Mb on chromosome 6. The thresholds are labeled with black dotted lines. The dashed red lines denote the SNPs ch06_41074832 and ch06_41338689. (C) Distribution of 15 alleles among Wild, PIM, CER, BIG, and *F*_1_ accessions. Different locus genotypes are marked by R, H, and A.

A previous report proposes a two-step evolution of fruit mass: domestication of *Solanum pimpinellifolium* (PIM) to *Solanum lycopersicum* var. *cerasiforme* (CER) and improvement of CER to *Solanum lycopersicum* (BIG) [23]. Whether the regulatory genes or loci of MA content in tomato fruits have been selected during domestication or improvement, the GWAS analysis enabled us to determine how MA content was domesticated. We next sought to dissect the selective sweeps of 15 candidate gene-association SNPs underlying domestication and improvement. *F*_ST_ and *π* values in the regions of 15 associated loci were analyzed in PIM, CER, and BIG accessions (Supplementary Data Tables S5 and S6), and visualizations of two genetic parameters are shown in Fig. 6A and B and Supplementary Data Figs S3 and S4. When comparing *F*_ST_ between PIM and CER accessions (Fig. 6A) or *F*_ST_ between CER and BIG accessions (Fig. 6B) at SNPs ch06_41074832 and ch06_41338689, none of the *F*_ST_ values exceeded the thresholds. However, *F*_ST_ values still showed an intermediate degree of differentiation between groups PIM and CER, and great genetic divergence between CER and BIG (Fig. 6A and B). In addition, we calculated nucleotide diversity in the comparison of PIM and CER (*π*_PIM_/*π*_CER_) as well as in the comparison of CER and BIG lines (*π*_CER_/*π*_BIG_). For *π*_PIM_/*π*_CER_ or *π*_CER_/*π*_BIG_ we did not detect domestication or improvement sweep signals in the genomic regions harboring SNPs ch06_41074832 and ch06_41338689. These data showed that no domestication/improvement selective sweep signals overlapped with significant major loci in region 40.8–41.59 Mb on chromosome 6. In addition, we also examined the selective sweeps of other 13 association SNPs. Only four SNPs (ch01_5153996, ch01_78818632, ch06_41008267, and ch09_60583005) were detected with domestication sweep signals with significant *π*_PIM_/*π*_CER_ values, and no improvement sweep signal was found (Supplementary Data Tables S5 and S6).

We analyzed the detailed allele distributions of 15 association SNPs in five tomato subpopulations (Fig. 6C). One of the more obvious findings was that the distribution of 15 alleles in CER and BIG showed no differences, indicating that these alleles were not selected in the improvement process. During the domestication stage, the T genotypes of SNPs chr01_5153996, chr07_63453334, and chr09_60583005 showed a drastic reduction in distribution. On the contrary, many SNPs, e.g. chr01_78818632, ch06_41074832, and chr06_45067556, exhibited significantly increased A distributions in CER relative to PIM. It was remarkable that type H of some SNPs maintained a relatively stable distribution in PIM, CER, and BIG, such as chr06_41008287, chr06_41338689, and chr08_53574022. In Wild accessions, only SNPs chr07_63453334, chr09_59796046, and chr06_41008267 possessed two or three genotypes; the remaining SNPs had only a single R genotype (low-MA genotype). Replacing undesirable alleles should have a strong positive effect on consumer preferences [21]. Modern commercial *F*_1_ hybrids obviously avoided genotype R in 15 SNP loci in our study (Fig. 6C). We detected the MA levels and 15 SNP genotypes of 24 modern cultivars (Supplementary Data Fig. S5). Only a few genotypes A have been found, whereas a large number of genotypes R existed. Interestingly, the proportion of genotype H was very high (especially major SNPs chr06_41008267 and chr06_41338689). It could be speculated that a high proportion of genotype H might be the breeding strategy to reduce the genetic effect of allele A by generating heterozygous loci.

**Figure 7:**
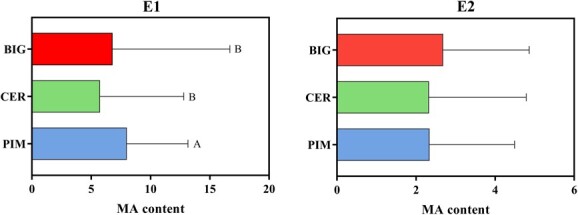
Distributions of MA content from different tomato accessions in E1 and E2. Different uppercase letters represent significant differences at *P* ≤ .001 by *t*-test.

To elucidate the causality between genotype and phenotype, we assessed the distributions of MA content within different tomato accessions in E1 and E2 (Fig. 7). The contents in CER and BIG showed no difference in E1 and E2. Under environment E1, the MA content of PIM accessions was statistically higher than those in CER and BIG. We were curious about why there was no phenotypic difference among three tomato groups under the E2 condition. The MS and SS loci obtained from different environment-based GWAS might provide further insight into the genetic basis of MA accumulation.

### Malic acid content varies with the different allele combinations

**Figure 8:**
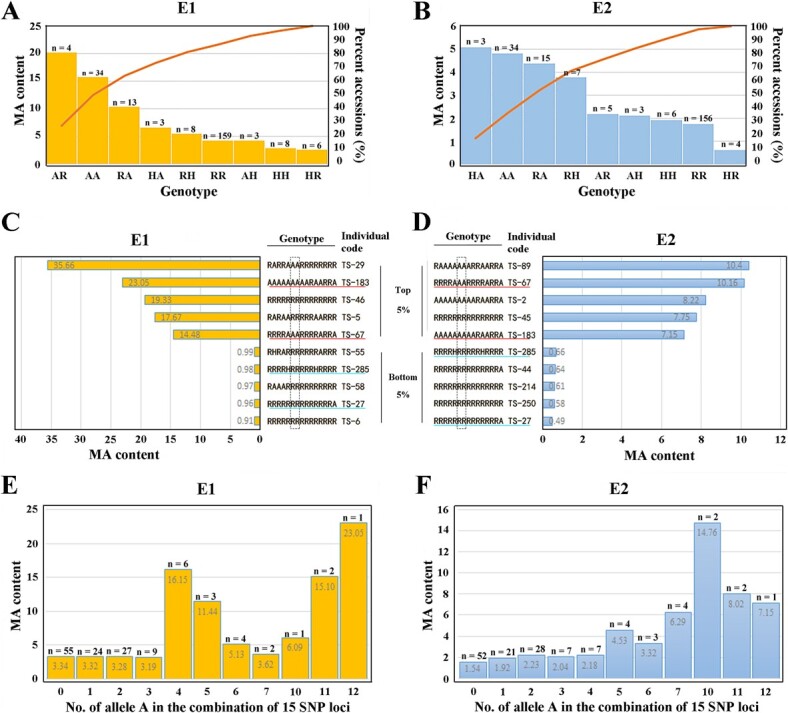
Combinations of alleles for tomato MA content. (A, B) Major SNP allele distribution of MA content at positions ch06_41074832 and ch06_41338689. The numbers of accessions for this genotype are indicated above the columns. (C, D) Genotypes (combinations of 15 candidate-gene-association SNP alleles) with the highest or lowest 5% MA content in 91 individuals with major loci AA/RR. The distribution order of the 15 SNP positions was ch01_5153996, ch01_78818632, ch04_2164254, ch04_2970001, ch06_41008267, ch06_41074832, ch06_41338689, ch06_43832543, ch06_45067556, ch07_63453334, ch08_53462894, ch08_53574022, ch09_59796046, ch09_60583005, and ch12_64414473. The SNPs marked in the dashed black boxes are two major SNPs (ch06_41074832 and ch06_41338689) in (A). The same samples with the top or bottom 5% MA content in both E1 and E2 are marked with red and green lines, respectively. The genotypes in 360 tomato accessions are listed in Supplementary Data Table S7. (E, F) Correlations between the number of alternative alleles A in the combination of 15 SNP loci and MA content. The numbers of accessions for this genotype are indicated above the columns.

Diverse tomato accessions produced a lot of alleles and presented very different allele combinations. Based on the MA content distributions (Fig. 4), allele combinations of 15 candidate-gene-associated SNPs contributed to MA content in each individual. We determined the MA distribution for allele combinations of two major SNPs, and nine combinations are shown in Fig. 8A and B. Combinations of homozygous genotypes (RR and AA) contained the largest number of individuals, and a significant MA content difference was found between genotypes RR and AA under both E1 and E2 environments. When any SNP in genotype RR was mutated to A, the MA content increased. Nevertheless, some genotypes had fewer individuals (e.g. AR, HA, and AH), leading to insufficient statistical power. Since genotypes AA and RR of major loci showed stable differential performance under repeat environments, we counted the 15 SNP complete genotypes of 91 individuals with major loci AA/RR (Fig. 8C and D). Under both E1 and E2 environments, TS-64/183 and TS-27/285 were the top and bottom 5%-value individuals, respectively. Regarding the incongruences, TS-46 and TS-45, possessing low-MA-content genotypes, were classified as the high-MA tomatoes in E1 and E2, respectively, but nonetheless carried 15 R alleles related to low MA content. The tomatoes with lowest 5% MA content had abundant R alleles associated with low MA concentration while being classified as low-MA individuals. With the increase in the total genotype A number in the combinations of 15 SNP loci, MA content was kept at a relatively high level (Fig. 8E and F). A weak correlation could be defined between allele A number and MA content. Based on the comparison of tomato MA content with different SNP genotypes (Fig. 4), it could be inferred that the theoretical genotype of high MA content was AAAAAAAAAAAAAAA (15A) and low MA accessions should be RRRRRRRRRRRRRRR (15R). In fact, genotype 15A did not exist in our population, and few individuals had more than 10 genotypes A (Fig. 8E and F). Type R alleles of associated SNPs were the most distributed alleles (Fig. 6C), and therefore a large number of 15R-genotype indiv iduals were found. This might explain the large number of low-MA-content tomato accessions in this population (Fig. 1A).

## Discussion

**Figure 9:**
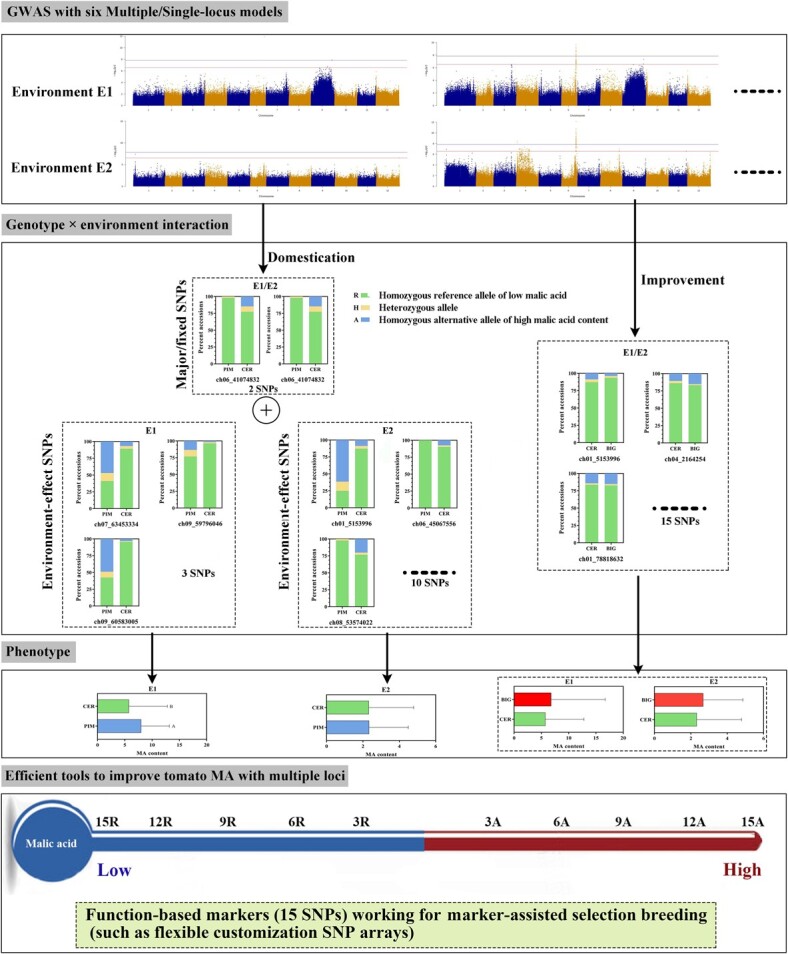
Workflow of the genome-based selection of loci for MA accumulation.

Breeders have been focusing on tomato improvement of commodity traits, such as yield, resistance, shelf life, and fruit color [24]. Tomato fruit weight was negatively correlated with major flavor substances, including acids (citrate, MA), sugars (fructose, glucose), and a range of volatile compounds [19]. This leads to substantially less flavor in modern commercial tomato varieties [21]. At present, food taste and nutrition have been the key concerns for consumers as well as breeders. MA not only plays a role as a mediator in a series of metabolic pathways, but is also an important flavor acid in fruits [2, 5, 10]. MA accumulation is determined by a complex regulatory network [9–11]. GWAS is a classical genetic approach to identifying QTLs and causal genes underlying breeding traits in crops [20]. In the present study, we performed a GWAS to explore the loci associated with MA content and discuss the potential genotype × environment interaction related to MA content (Fig. 9).

### Advantage of GWAS using multi-models and repeated environment

To completely detect the SNP variations associated with MA content, GWAS were carried out based on two environments. A major issue in model selection is controlling false positives or false negatives [25]. Thus, six multiple-/single-locus models were used to identify associations between SNPs and phenotypes that ranged from simple to increasingly complex. We obtained suggestive or associated SNPs collected in repeat-environment GWAS results aggregated from different models (Supplementary Data Tables S2 and S3). Identification of candidate genes is one of the main goals of GWAS. Potential candidate genes associated with SNP loci play the roles of central regulators for MA metabolism pathways or determine rate-limiting catalysis or transport [26]. In this study, we identified 15 promising candidate genes, which could be divided into three types, MT, MS, and SS, and association loci of almost half of the genes have been previously reported at least once (Table 1) [3, 10, 19–22]. MT-type loci were detected in both environments, and the TFM6 loci were identified by many GWAS studies of MA content. This property suggested an important role of the 15 candidate genes in the accumulation of MA [6, 13]. The candidate *SlALMT9* gene has been identified for its role in MA accumulation [10, 13]. But another point of view implied that its contribution to MA content may be less affected by the environment. Moreover, the lead SNP of TFM6 in our study was located exactly at the exon of the *SlALMT9* gene (Fig. 3), and was closer to the causal gene than in other studies [3, 10]. In addition, the physical distance between TFM6.2 and TFM6 was 264 kb on chromosome 6 and their lead SNPs belong to independent LD blocks (Fig. 3). The −log10(*P*) values of their lead SNPs showed significant differences in different model–environment combinations (Supplementary Data Fig. S6). In addition, premature termination of the gene coding region is also the type of mutation that leads to structural changes in the protein after translation [21]. Moreover, another multiple-locus model, SUPPER, was used to test lead SNPs (Supplementary Data Fig. S7). The correlation results showed that lead SNPs ch06_41074832 and ch06_41338689 were consistently detected in E1 and in E2, respectively. We believe that these two lead SNPs are associated with independent loci underlying MA content. MS-type SNPs were obtained using GWAS with several models, which indicates a moderate level of confidence. Previous studies have proved that there is a relationship between these loci/genes and MA content [20, 21]. SS-type SNPs were only detected in a single model with one environment, which indicates a weak level of confidence. Interestingly, SS-type loci TFM6.5 and TFM8.2 have also been shown to be significantly associated with MA content in another study [21].

### Flexibility of causality between genotype and phenotype

A previous study reported that the MA content showed a wide range of variation [20]. It is of interest to speculate about the effect of human selection on MA content in tomato breeding. In our study, the MA content displayed no obvious phenotype difference between CER and BIG lines in 2-year repeats (Fig. 7). Based on the genetic parameters of population differentiation, no sweep signal was found during the improvement stage among the genomic regions of 15 SNPs associated with MA content (Fig. 6A, Supplementary Data Table S6). Moreover, these SNP alleles also showed similar distributions between CER and BIG accessions (Fig. 6C). This evidence is not suggestive of an improvement event. The same and different results have been found in other studies. In a GWAS population containing 398 chemically profiled accessions, no significant MA difference was found in ripe fruits between CER and BIG accessions [21] (Supplementary Data Fig. S8A). Even in repeated trials, the resulting phenotypes may differ due to environmental changes. MA data from 144 BIG samples differed from those of 104 CER samples in environment ZD, but showed no difference in environment HD [10] (Supplementary Data Fig. S8B). Similar results have been shown in other studies, where the MA content of CER and BIG showed completely opposite variations in two repeated experiments (Supplementary Data Fig. S8B) [3]. It is clear that phenotypic differences between CER and BIG accessions (tomato improvement stage) in our study are also being demonstrated by selective sweep analysis and SNP distribution.

For selection during the domestication stage, the MA content of CER tomatoes in E1 was lower than that of PIM, while the MA content of CER tomatoes in E2 was similar to that of PIM (Fig. 7). Some studies under different cultivation conditions revealed a substantial environmental effect [27–29]. We speculate that both environmental and genotypic variation between trials contributed to the phenotype difference in our study. MT-type loci (major) were detected from the GWAS results in E1 and E2 (Table 1). Two MS-type SNPs (ch09_59796046 and ch09_60583005) and one SS-type SNP (ch07_63453334) were associated with MA content in E1, and it was interesting that the low-MA-content genotype R of these three SNPs showed a drastic increase during domestication selection. Furthermore, the MS-type SNP ch09_60583005 and another SNP, ch01_5153996 (the proportion of genotype R also increased during domestication), overlapped with chromosome regions of the domestication selective sweeps (Supplementary Data Table S6). These results reveal that the MA content of CER in the comparison with PIM is reduced in environment E1, possibly caused by the genetic effects of these associated SNPs identified from E1 (Table 1). Turning attention to E2, the modest increase in proportions of genotypes A were explored in CER accessions compared with PIM among all association SNPs (except chr01_5153996) acquired from environment E2 (Fig. 6B). However, none of these SNPs overlapped with those identified in E1, suggesting that multiple genes subject to environmental regulation underlie the MA trait. These results are in full agreement with the observed difference between E1 and E2 during domestication. Thus, we believe that the tomato MA content is more influenced by domestication sweeps and less affected by improvement sweeps in long-term breeding (Fig. 9).

Environmental factors play an important role in the regulation of fruit secondary metabolism [30]. The genetic background determined the MA concentrations of PIM, CER, and BIG tomato fruits, whereas environmental factors cause prominent quantitative changes in MA. Temperature, nutrients, water, light, and biotic/abiotic stresses have been shown to affect the production of secondary metabolites in fruits [31, 32]. Two years of repeated operation reflect the phenotype difference in subgroups with diverse backgrounds under the challenge of environmental factors. The current GWAS identified key loci regulating MA content in tomatoes with diverse genetic backgrounds in repeat environments. Further, identification of the specific loci responding to environmental regulation is very meaningful for revealing the genotype × environment interaction related to the formation of MA (Fig. 9). Loci in different environments may differ in their ability to contribute to phenotypes, which may be one explanation for the differences in phenotypic data under different environments.

### Efficient tools to improve tomato MA with multiple loci

Techniques such as genotyping, marker-assisted selection, and genomic selection can be used for rapid breeding [26, 33]. Marker-assisted selection has been successfully applied in the breeding of almost all crops. Whole-genome high-throughput genotyping platforms, genotyping by sequencing, array-based genotyping, and PCR-based markers are vital for marker-assisted breeding [34]. However, the biggest challenge of genotyping is its cost. Next-generation sequencing technologies are still expensive and the use of PCR-based markers is also a laborious and time-consuming genotyping method. The application of economical and rapid breeding programs is essential. Array-based genotyping platforms are a cost-effective alternative and provide the flexibility to customize detection probes. To date, high-density SNP genotyping arrays have been designed and used in marker-assisted breeding of crops [34, 35].

A previous study developed an indel_3-based CPAS marker to genotype the MA content in tomato fruit [10]. Complex traits, such as MA, are affected by large numbers of variants with small effects [3, 10, 18, 19]. The identification of MA-associated variation at the whole-genome level is vital for breeding for MA customization. In our study, 149 suggestive/significant and 15 candidate gene-association SNPs related to MA content are available to develop association-based markers (association SNPs) and function-based markers (gene SNPs), respectively. If the SNPs are in the annotated genes or promoter regions, the candidate genes are high-confidence annotated genes usable for gene SNP analysis [34]. Additionally, the number and percentage of SNPs located in intergenic intervals and associated genes are important to measure the quality of SNP chips. Thus, gene function-based markers of MA content are required for marker-assisted selection breeding, and our study provides key SNP information for the development of high-quality, low-cost, high-throughput, and flexible customization SNP arrays (Fig. 9).

## Materials and methods

### Plant materials and genomic information

A previous study outlines the histories of tomato domestication and improvement with a classical population containing 360 tomato accessions [23]. In our study, this natural population, including 53 *Solanum pimpinellifolium* (PIM), 112 *S. lycopersicum* var. *cerasiforme* (CER), 171 *S. lycopersicum* (BIG), 17 modern commercial hybrids (*F*_1_), and 10 wild tomato accessions (Wild, 1 *Solanum habrochaites*, 3 *S. cheesmaniae*, 1 *S. galapagense*, 3 *S. peruvianum*, 1 *S. neorickii* and 1 *S. chilense*), were used to identify the distribution of SNP variants and analyze the genetic parameters of population differentiation.

Genomic information on 360 tomato accessions population is available from the National Center for Biotechnology Information Sequence Read Archive (accession number SRP045767) [23]. All paired-end sequence reads were aligned against the tomato reference genome (release SL2.40) using BWA software [36], and SNP calling was performed with the software Genome Analysis Toolkit [37] under default parameter values. Subsequently, a variant dataset based on genotyping-by-sequencing was filtered with a minor allele frequency <5% and missing rate <20%. Finally, a subset of 3 535 044 SNPs across the 360 tomato accessions was obtained and used for further analyses. Individual heterozygosity was calculated using PLINK version 1.9 [38].

### Phenotypic evaluation

All materials were planted at Huazhong Agricultural University, Wuhan, China, in 2013 (open field) and 2016 (greenhouse). In the greenhouse environment, plants were grown at 25 ± 2°C with 70% relative humidity under a 16 hours/8 hours day/night photoperiod. Each sample contained at least three plants with at least three ripe fruits per plant. The samples were frozen with liquid nitrogen, then stored at −80°C for the measurement of MA level. The MA content was presented as in our previous study [10, 39]. Reliable phenotypic data on 266 accessions in E1 (1 Wild, 20 PIM, 102 CER, 129 BIG, and 14 *F*_1_) and 260 accessions in E2 (3 Wild, 22 PIM, 94 CER, and 141 BIG) were obtained for constructing association mapping panels. R package lme4 was used to calculate the broad-sense *H*^2^ [40].

### Description of association mapping models

The association analyses were performed using six models of association mapping including: Mixed Linear Model (MLM) [41], General Linear Model (GLM) [42], Compression MLM (CMLM) [43], Multiple loci Mixed Model (MLMM) [53]. Fixed and random model Circulating Probability Unification (FarmCPU) [44], and Bayesian Information and Linkage-disequilibrium Iteratively Nested Keyway (BLINK) [38]. Models GLM, MLM, and CMLM are single-locus models, and models MLMM, FarmCPU, and BLINK are multi-locus models. In terms of statistical power, multiple-locus models (MLMM, FarmCPU, and BLINK) are superior to others [45]. Within the single-locus model category, CMLM is superior to MLM, and MLM is superior to GLM. Within the multiple-locus model category, BLINK is superior to FarmCPU, and FarmCPU is superior to MLMM [45].

### Association mapping

Based on genome-wide SNPs, six association mapping models were used to calculate the correlation between a SNP locus and the MA trait. GWAS was conducted by *R* package GAPIT (version 3) using the association mapping models described above [45]. The first three principal components of a principal component analysis were used to control population structure for all six models. The genetic relatedness was calculated using the VanRaden kinship matrix [46]. The genome-wide significance threshold was set to suggestive (1/*n*, where *n* is the effective number of independent SNPs, 2.83 × 10^−7^) and significant (0.05/*n*, 1.41 × 10^−8^) *P*-value thresholds to define the significant association SNP loci. The effective number of independent SNPs was calculated using Genetic type 1 Error Calculator software [47]. The R package qqman was used to visualize the GWAS results (Manhattan plots and QQ plots) [48].

### Characterization of candidate genes

All significant SNPs (*P* < 1.41 × 10^−8^) obtained from multi-model GWAS across the two environments were used to identify potential candidate genes regulating MA accumulation. The physical locations of SNPs were determined based on the tomato reference genome version SL 2.40, and candidate genes were annotated according to their corresponding ITAG2.3 annotation information (http://solgenomics.net/). LD was visualized and haplotype blocks were constructed using the LDBlockShow software [49], and the correlation coefficient (*R*^2^) was calculated to determine pairwise LD decay. SNPs showing high levels of LD with the peak SNP (*R*^2^ = .6–1) were considered to be in LD. Peak SNPs in LD regions were used to predict candidate genes. If these peak SNP variants were directly located at the annotated genes or promoter regions (2 kb before the gene), the genes and SNP variants were thought to be associated with MA content. Subsequently, we performed comparative analyses of MA content in accessions with different SNPs of candidate gene association, and further excluded the candidate genes obtained from inaccurate lead SNPs.

### Expression patterns of candidate genes

We further cross-checked the expression patterns of candidate genes. Gene expression in different organs and fruits at different stages in tomatoes with three genomic backgrounds was studied, including *S. lycopersicum* cv. ‘Micro-Tom’ [50], *S. pimpinellifolium* LA1589, and *S. lycopersicum* cv. ‘Heinz’ [downloaded from the Tomato Functional Genomic Database (http://ted.bti.cornell.edu)]. Fruit spatiotemporal expressions of candidate genes were also provided in another profile with the transcript of cultivated variety *S. lycopersicum* cv. ‘M82’ [51]. HemI software was used to visualize the gene expression patterns with heat maps and hierarchical clustering (http://hemi.biocuckoo.org/down.php).

### Selective sweep detection during domestication and improvement

Nucleotide diversity (*π*) is often used as a measure of the degree of variation in a population [3, 23]. The fixation index (*F*_ST_) is also applied to confirm highly differentiated regions of molecular diversity [10]. Analyses of *π* and *F*_ST_ were performed to identify the selective sweeps about MA content at two key stages (domestication and improvement) in tomato evolution [23]. These selective sweep signals were selected during the domestication or modification stages. The parameters were calculated using the VCFtools package [52] with a 100-kb sliding window and a step size of 10 kb for genome-wide scanning or a 10-kb window with a step size of 1 kb for the 0.79-Mb interval on chromosome 6 (40 800 000–41 590 000). The windows with the top 5% of genetic diversity ratios between PIM and CER (*π*_PIM_/*π*_CER_) as well as between CER and BIG (*π*_CER_/*π*_BIG_) (2.20 and 7.65 for domestication and improvement, respectively), and population differentiation ratios *F*_ST_ (PIM versus CER) and *F*_ST_ (CER versus BIG) (0.60 and 0.14 for domestication and improvement, respectively) were identified as selective sweep regions.

## Acknowledgements

This work was supported by grants from the National Key Research & Development Plan (2022YFD1200502; 2021YFD1200201); the National Natural Science Foundation of China (31972426; 31991182); the Wuhan Biological Breeding Major Project (2022021302024852); the Key Project of Hubei Hongshan Laboratory (2021hszd007); the Hubei Key Research & Development Plan (2022BBA0062; 2022BBA0066); the Fundamental Research Funds for the Central Universities (2662022YLPY001) and the International Cooperation Promotion Plan of Shihezi University (GJHZ202104)..

## Author contributions

W.G. and Y.Z. conceived and designed the experiments. W.G. and F.Y. analyzed the data and carried on the analyses. J.T. and X.Z. performed the data collection. S.H., Yaru W., Ying W., F.Y., L.Y., L.S., F.L., P.G., F.W. and H.D. conducted the literature review and edited the manuscript. Y.Z. was responsible for funding acquisition and article revision.

## Data availability

The datasets supporting the findings in this study are available in the supplementary materials.

## Conflict of interest

The authors declare no competing interests.

## Supplementary data

Supplementary data is available at *Horticulture Research* online.

## Supplementary Material

Web_Material_uhad021
