# Supplementary material for: Multiple-model GWAS identifies optimal allelic combinations of quantitative trait loci for malic acid in tomato
Source: Hortic Res. 2023 Feb 14;10(4):uhad021. doi: 10.1093/hr/uhad021 (PMC10076212; doi:10.1093/hr/uhad021)
Supplement: Web_Material_uhad021 [file web_material_uhad021.zip › Supplementary materials.docx]

**Supplementary materials**


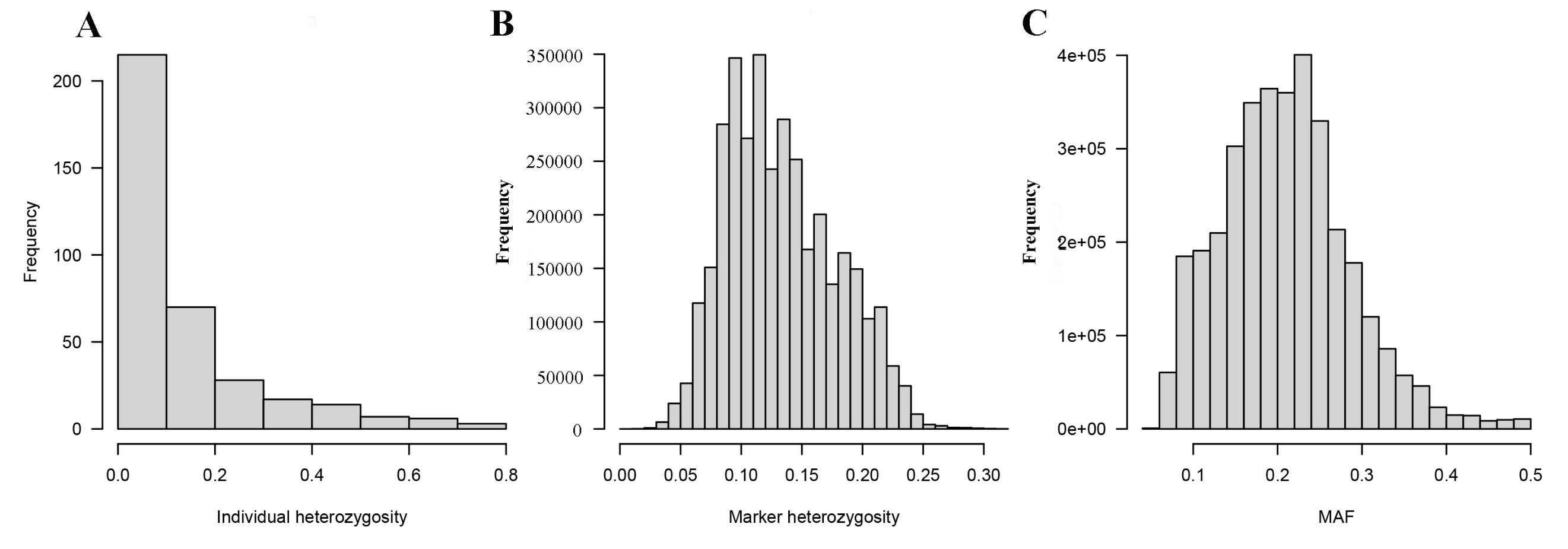


**Supplementary Fig. S1** The frequency of individual heterozygosity (A), marker heterozygosity (B), and MAF (C).


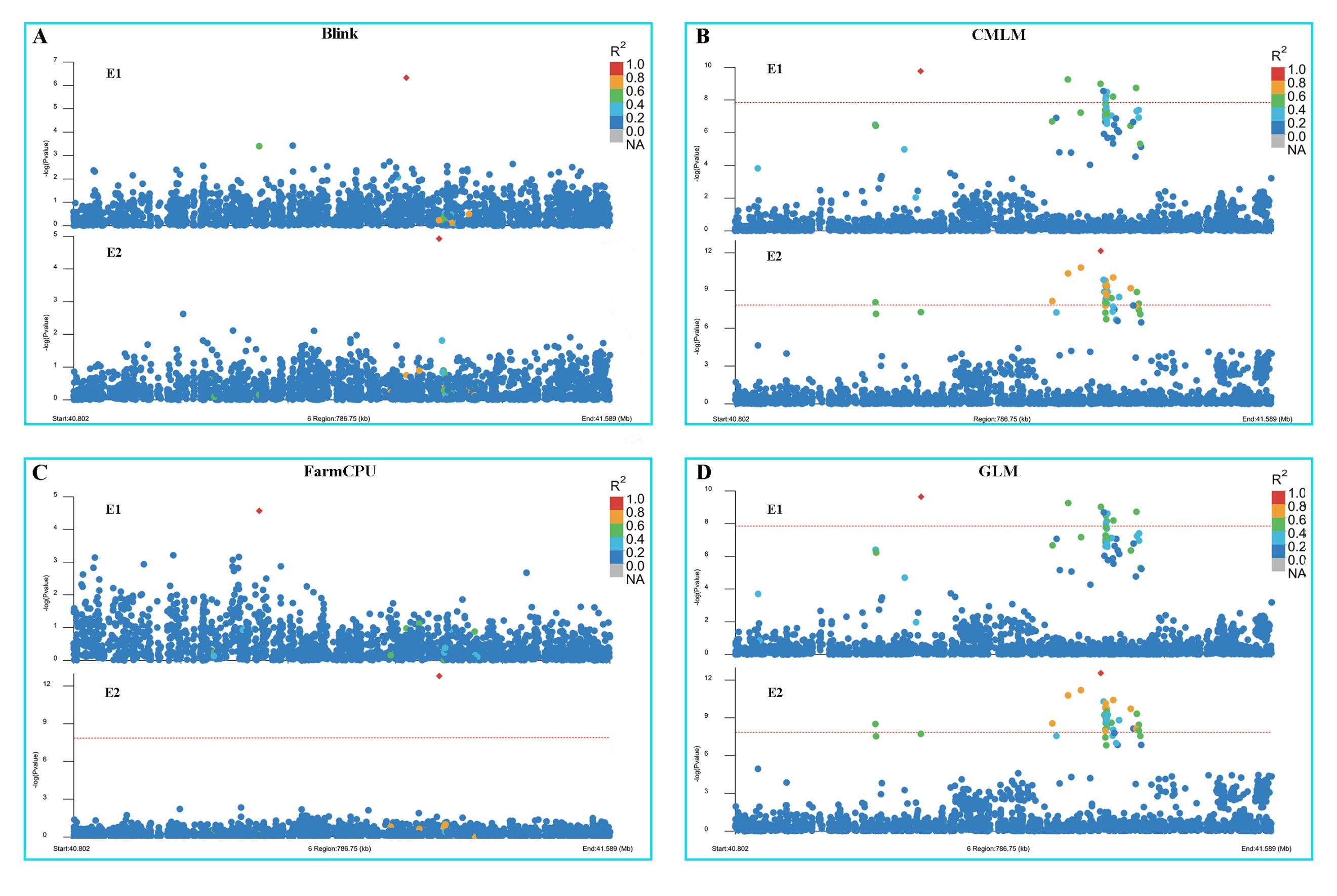


**Supplementary Fig. S2** Detailed plots from GWAS results in region 40.8 – 41.59 Mb on chromosome 6 (x-axis).


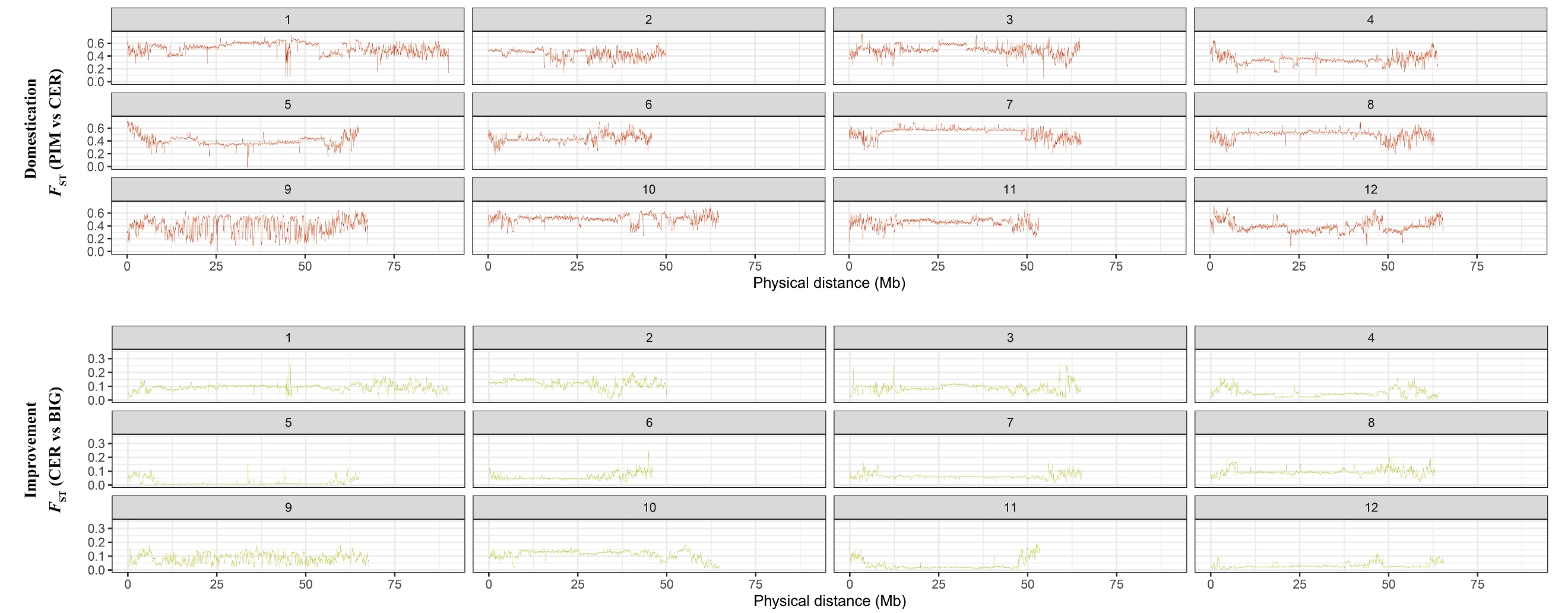


**Supplementary Fig. S3** *F*_ST_ values for all SNP sites during domestication and improvement.


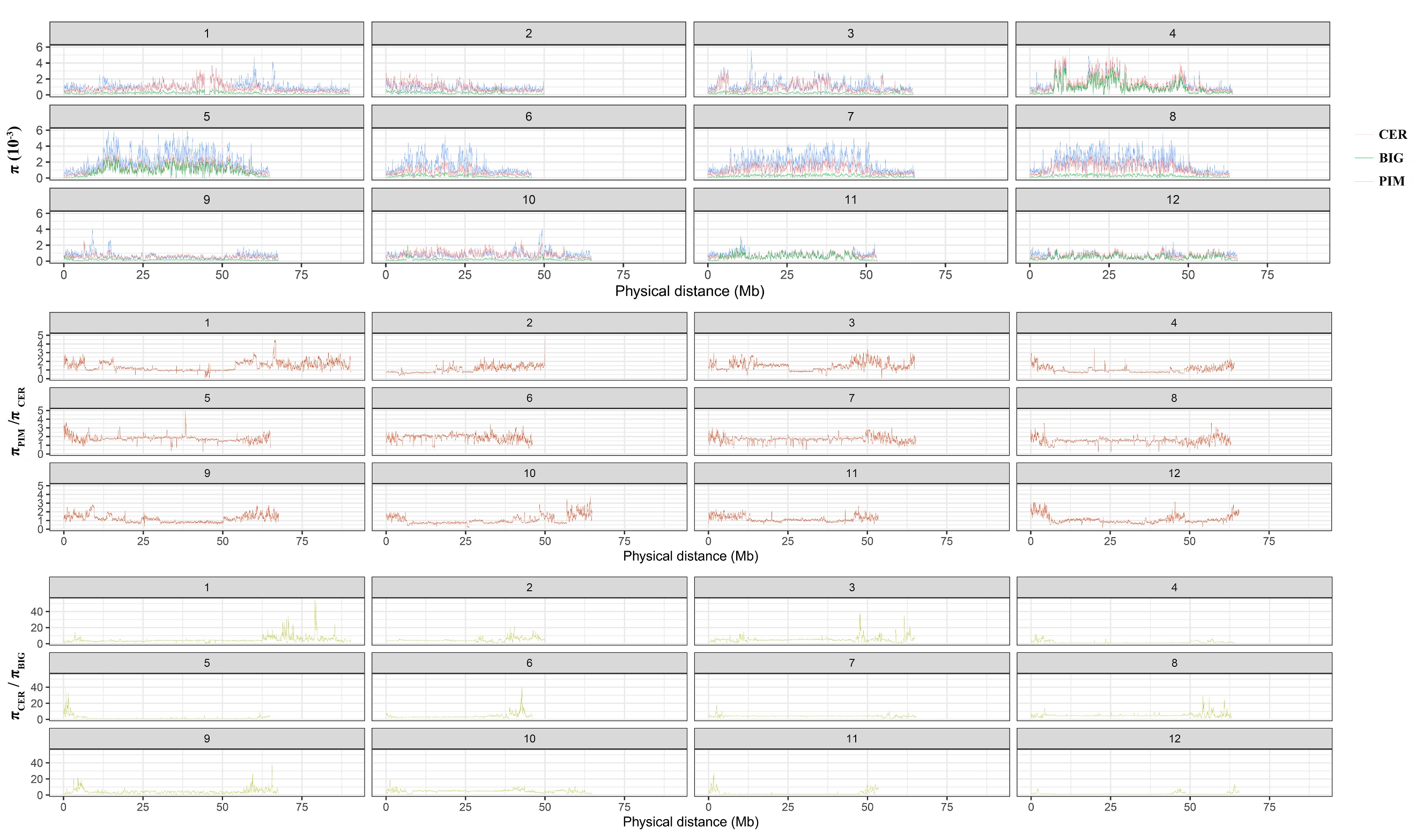


**Supplementary Fig. S4** π and π ratios values for all SNP sites during domestication and improvement.


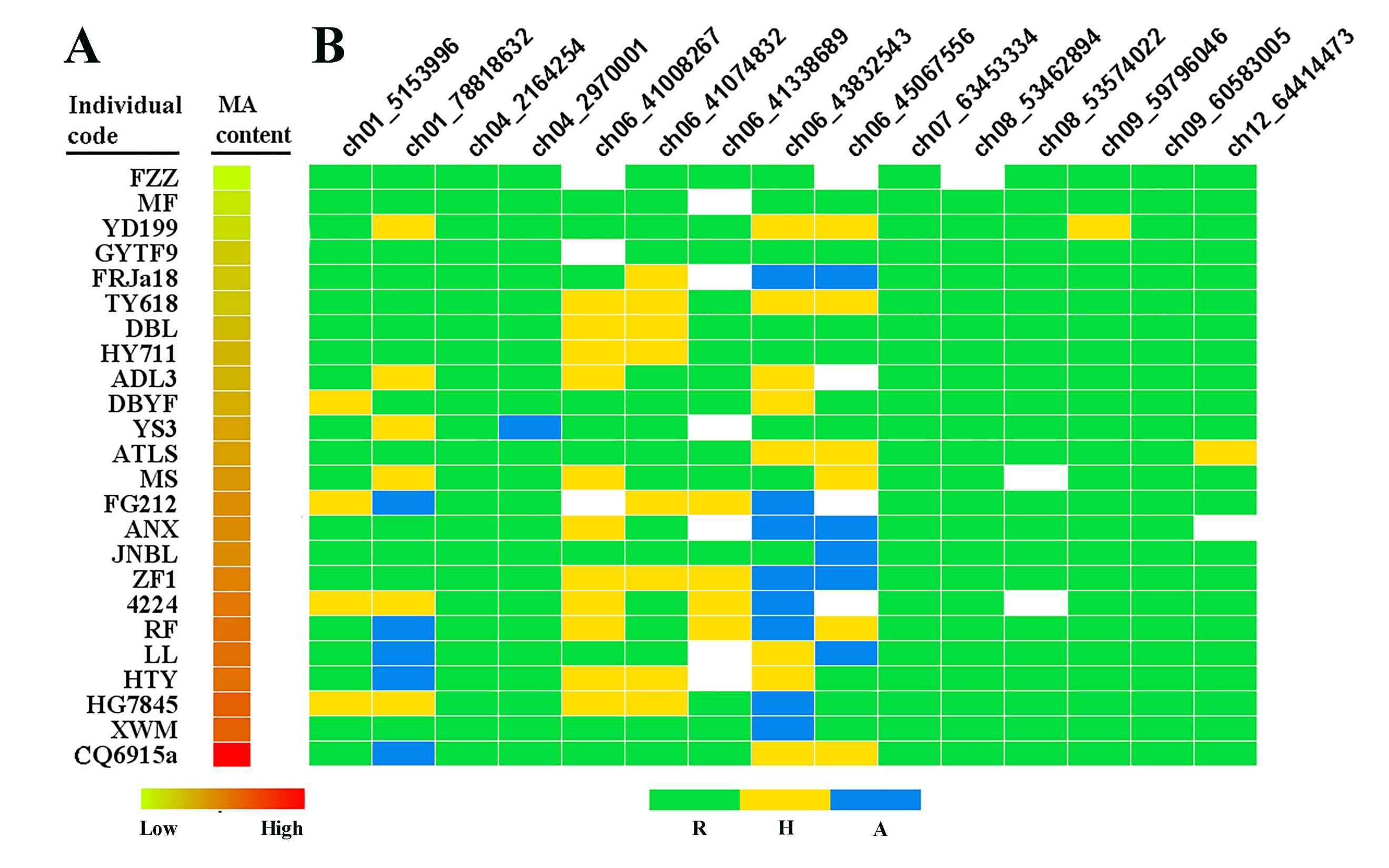


**Supplementary Fig. S5** The MA levels (A) and 15 SNP genotypes (B) of 24 modern breeding variety. The white boxes represent vacant genotypes.


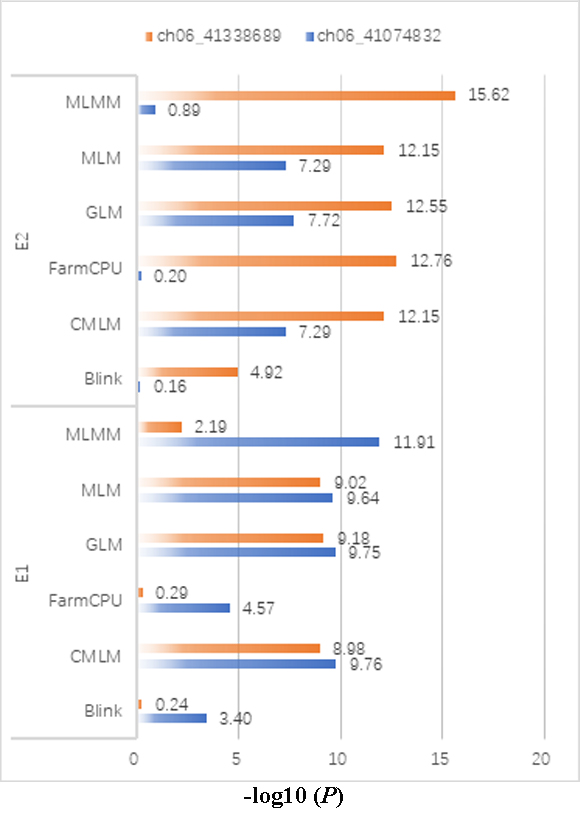


**Supplementary Fig. S6** The -log10 (*P*) values at major SNPs in different model-environment combinations.


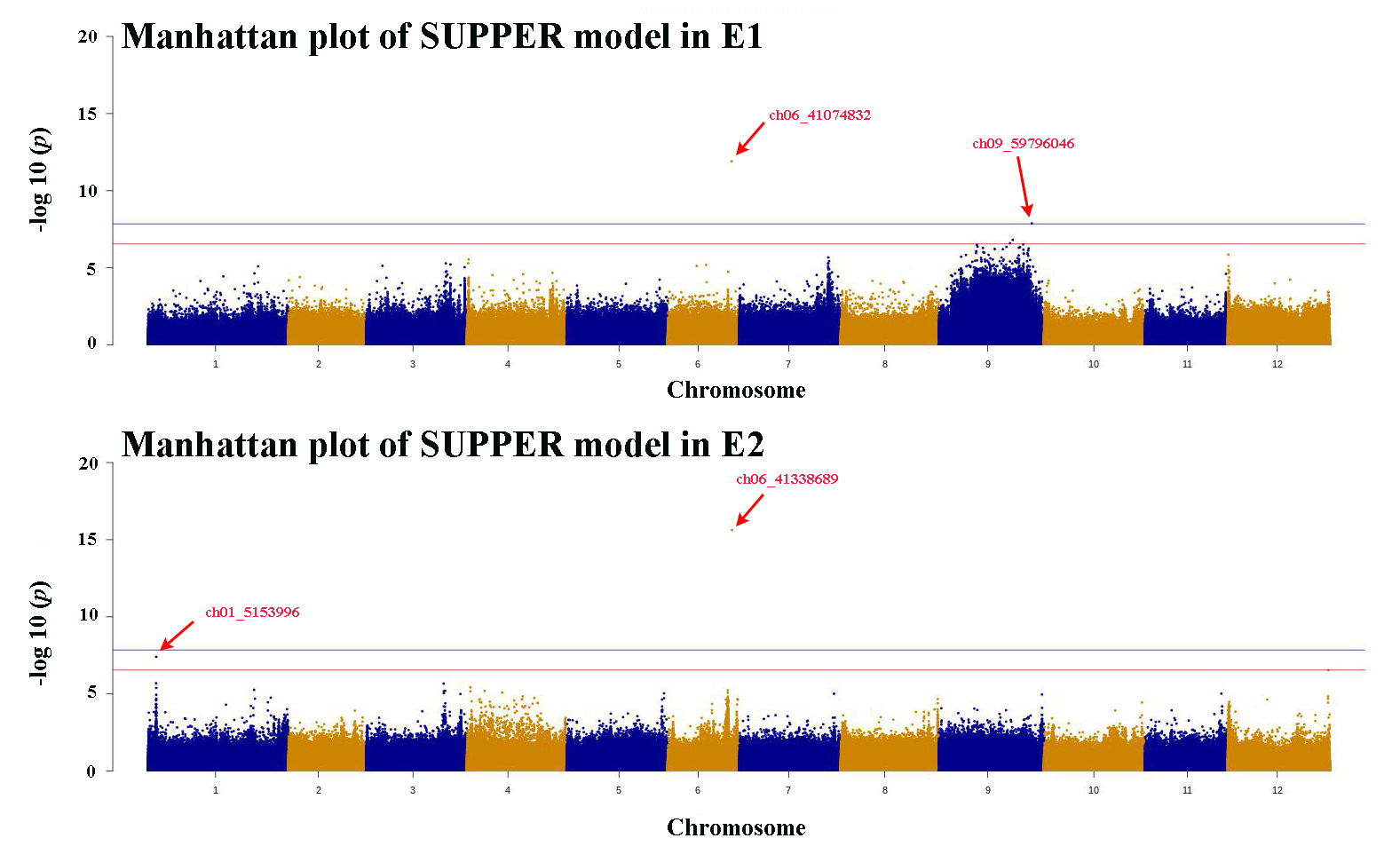


**Supplementary Fig. S7** Manhattan plots with SUPPER model in E1and E2. SUPPER model is carried out using GAPIT software (<http://zzlab.net/GAPIT>). −log10 (*P*) values from the GWAS results are plotted on the y axis. The blue lines indicate genome-wide significant threshold 7.85, and the orange lines indicate suggestive threshold 6.55. The SNPS associated with candidate genes are marked in Manhattan plots by red dotted lines.


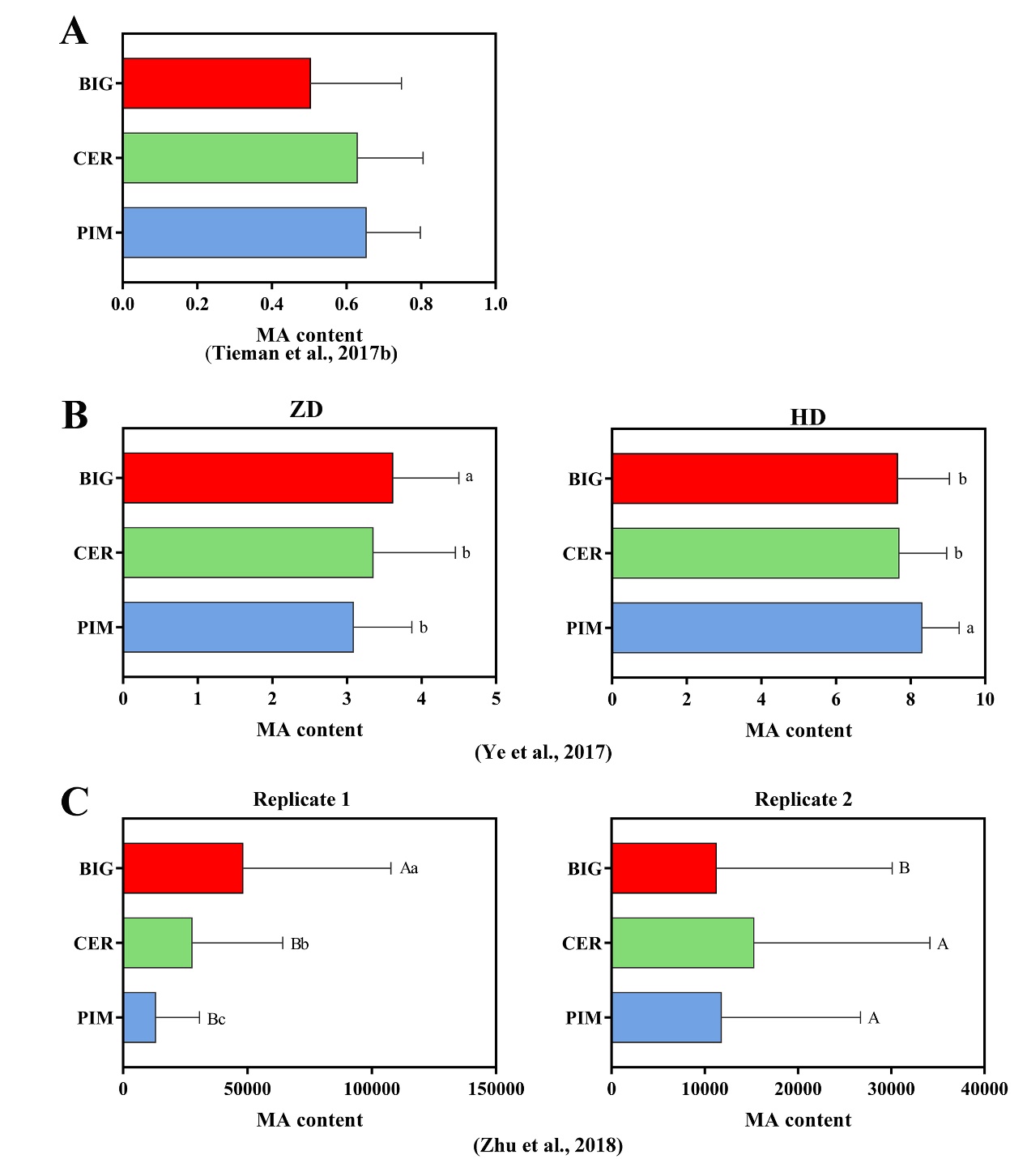


**Supplementary Fig. S8** Distributions of MA content from different tomato accessions in previous studies. Citations are indicated at the bottom of the picture. Different uppercase or lowercase letters represent significant differences at *p* ≤ 0.001or 0.05 by *t* test, respectively.

**Supplementary Table S1** Summary of 360 tomato accessions and malic acid (MA) content detected in two different environments.

**Supplementary Table S2** The summary of all significant SNPs identified by genome-wide association analysis. For each type, SNP, chromosome, position (bp), reference allele (Ref), alternative allele (Alt), environment, model, −log10 (*P*), candidate gene, variation position, associated gene, and annotation are provided. Multiple models and double environments (MD), multiple models and single environment (MS), single model and single environment (SS).

**Supplementary Table S3** All significant SNPs identified by multiple models and two environments. For each environment, model, SNP, chromosome, position (bp), *P*-value, minor allele frequency (MAF), R^2^ of the model without the SNP, R^2^ of the model with the SNP, and adjusted *P*-value are provided.

**Supplementary Table S4** Comparison of the number of significant SNPs identified by multiple GWAS methods.

**Supplementary Table S5** *F*_ST_ values of the flanking regions of SNPs associated with putative genes during domestication and improvement.

**Supplementary Table S6** π values of SNPs associated with putative genes during domestication and improvement.

**Supplementary Table S7** The genotypes of candidate genes-related SNPs in 360 tomato accessions. R represents homozygous reference allele. A represents homozygous alternative allele. H represents heterozygous allele. 0 represents the deletion of allele.
